# Front-of-Package Food Labeling to Reduce Caries: Economic Evaluation

**DOI:** 10.1177/0022034520979147

**Published:** 2020-12-17

**Authors:** M. Jevdjevic, S.R.W. Wijn, A.L. Trescher, R. Nair, M. Rovers, S. Listl

**Affiliations:** 1 Department of Dentistry—Quality and Safety of Oral Healthcare, Radboud University Medical Center, Radboud Institute for Health Sciences, Nijmegen, The Netherlands; 2 Department of Operating Rooms, Radboud UMC, Nijmegen, The Netherlands; 3 Department of Conservative Dentistry, Translational Health Economics Group, Heidelberg University, Heidelberg, Germany; 4 Department of Health Evidence, Radboud UMC, Nijmegen, The Netherlands

**Keywords:** diet, prevention, sugar consumption, dental public health, nutrition policy, decision making

## Abstract

Front-of-package food labeling (FoPFL) is increasingly advocated as an effective intervention to facilitate behavior changes toward healthier food purchasing and consumption, particularly in relation to products with added sugar. The present study assessed the potential caries-related impacts of FoPFL, using Germany as an example. The outcomes of interest were caries lesions prevented, dental treatment costs avoided, productivity loss reductions, and disability-adjusted life years (DALYs) averted. The baseline consumption of added sugar was derived from the German National Nutrition Survey. The reduction in sugar intake due to FoPFL was modeled according to estimates from a recent meta-analysis. Microsimulations were performed for 500,000 individuals and over a time horizon of 10 y. Deterministic and probabilistic sensitivity analyses were performed to check the robustness of results. For the period from 2017 to 2027, FoPFL was identified to prevent 2,370,715 (95% confidence interval [CI], 2,062,730–2,678,700) caries lesions and avert 677.62 (95% CI, 589.59–765.65) DALYs. Treatment cost savings amounted to €175.67 million (95% CI, €152.85–€198.49), and productivity losses reduced by €27.33 million (95% CI, €23.78–€30.88). Sensitivity analyses showed that the magnitude of the effects is highly dependent on consumers’ response to FoPFL. Our findings suggest that FoPFL has the potential to substantially reduce caries increment, caries-related morbidity, and economic burden. In addition, our study allows for the inclusion of oral health estimates in overall health estimates for sugar-related food labeling. Before prioritizing a strategy to tackle sugar consumption, decision makers should carefully consider all relevant context-specific factors and implementation costs.

## Introduction

According to the Organization for Economic Co-operation and Development (OECD), the overall market consumption of sugar is set to increase from 17.6 million tons in 2019 to 20.3 million tons in 2028 (Organisation for Economic Co-operation Development/Food and Agriculture Organization [OECD/FAO] 2019). For the period from 2018 to 2028, the average world level of per capita consumption is expected to increase from 22.7 kg/cap to 24.2 kg/cap. The need to reduce the intake of sugars for improving the health of people and communities is widely recognized (World Health Organization 2015). The World Health Organization (WHO) recommends restricting the intake of sugars, considering that they increase the body weight and the incidence of dental caries. Increased body weight is associated with noncommunicable diseases (NCDs), particularly diabetes and cardiovascular disease (Micha et al. 2017). Caries continues to be the most prevalent of all 300+ diseases and conditions assessed by The Global Burden of Disease Study (GBD; Kassebaum et al. 2015) and therefore deserves special attention.

In Germany, for example, caries has substantial economic impacts (Righolt et al. 2018) and detrimentally affects people’s quality of life (Walter et al. 2011). The caries-related costs attributable to the intake of free sugars surpassed US$5.67 billion in 2010 (Meier et al. 2017). Although the cumulative caries experience of the German population has been decreasing during recent decades, it remains substantial with a projection of 740 million decayed, missing, filled teeth (DMFT) in 2030 (Jordan et al. 2019). Dental caries is also a disease that accumulates over a lifetime resulting in more significant functional deficits in the latter part of life (Matsuyama et al. 2019). This has led to the assessment of potential pathways for caries development, and over the years, several contributing factors have been identified (Pitts et al. 2017). In all these pathways, sugars play a pivotal role, as shown by early studies that assessed very high levels of sugary food intake (Krasse 2001) and more recent studies that have shown a direct effect of sugars in causing dental caries (Moynihan 2016).

Food labeling is a frequently proposed strategy for reducing the amount of sugar intake (Kanter et al. 2018). It seeks to convert nutritional information into informed consumer choices toward healthy food and beverage consumption. Front-of-package food labeling (FoPFL) has been endorsed or adopted in more than 30 countries (Kanter et al. 2018; Jones et al. 2019). There are several FoPFL efforts, for example, a summary nutrient score, a traffic light system, a health logo, or warning labels (Kanter et al. 2018) (see Appendix for details). Such labeling was found to be effective in reducing the amount of food intake and incite reformulations of food and drinks (Shangguan et al. 2018).

In a recent policy statement of the International Association for Dental Research (IADR) and the American Association for Dental Research (AADR) on sugar-sweetened beverages (SSBs), one of the objectives for future research was to investigate the effectiveness of policies affecting marketing and advertising of these products (IADR/AADR 2020). Currently, there is no estimate of the potential oral health benefits due to food labeling. Therefore, the purpose of this study was to estimate the impact of food labeling on dental caries and its sequelae.

## Methods

**Figure 1.**
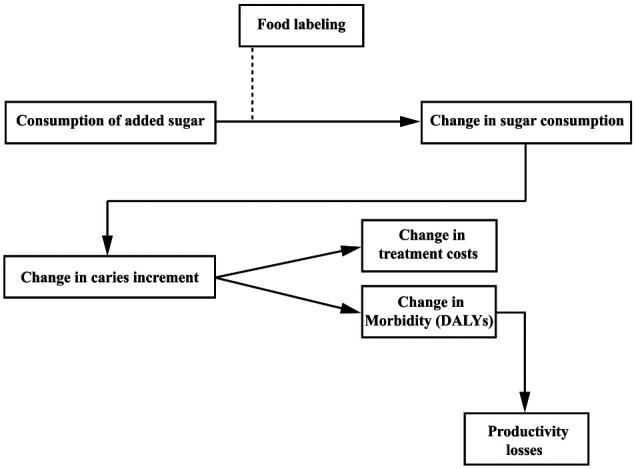
Conceptual framework of food labeling and its effects. DALY, disability-adjusted life year.

A decision-analytical microsimulation model was developed to estimate the potential caries-related impacts of FoPFL from a societal perspective in Germany. We considered any type of FoPFL labels providing the nutrition content or health-related information through logos, symbols, icons, or claims on the front of a package. It was assumed that the nutrition label would be presented on all products with added sugar. The impacts on oral health benefits and dental treatment costs were compared to a status without such a policy. The conceptual framework of our analysis is presented in Figure 1. The 2017 German population was used as a reference case.

### Model Structure

The microsimulation model consists of 2 primary health states: “no caries” and “caries.” All individuals start in the “no-caries” health state. In yearly cycles, every individual has a probability to develop caries. In the model, we assumed that each newly developed caries lesion was diagnosed at the first upcoming dental visit and a restoration was placed. Thereafter, patients returned to the no-caries state or had a probability to develop a new caries.

### Time Horizon and Outcomes

In line with previous work on sugar-directed policies (Schwendicke et al. 2016; Sowa et al. 2019), the simulations were performed over a 10-y time horizon to allow comparability. The outcomes of interest were caries lesions prevented, caries-related treatment costs avoided, disability-adjusted life years (DALYs), and productivity losses averted.

### Input Parameters

A person-level yearly caries incidence, stratified for age and gender, was derived from the publicly available online platform of the Institute for Health Metrics and Evaluation (IHME 2019). For treatment costs, the base case model incorporated a conservative value of €74.10 for a 1-surface restoration. Sensitivity analyses accounted for potentially higher treatment costs due to patient copayments and restoration failures. More details about the input values and data sources are available in the Appendix.

Baseline intake of free sugars was obtained from the German National Nutrition Survey II (NVS II). Data were available for the population aged 14 to 79 y (Appendix Table 1), stratified for gender and age groups (15 to 18, 19 to 24, 25 to 34, 35 to 50, 51 to 64, and 65 to 80 y old) (Heuer 2018). These aged categories were retained for the rest of the analyses.

**Table.** Number of Prevented Caries Lesions and Treatment Costs Avoided Due to Food Labeling with 95% Confidence Intervals, 10-y Time Horizon.

Characteristic	Caries Lesions Prevented (Total)	Treatment Costs Avoided (Million €)
Men		
Men aged 15–18	89,638 (80,984–98,292)	6.64 (6.00–7.28)
Men aged 19–24	152,929 (137,883–167,974)	11.33 (10.22–12.45)
Men aged 25–34	264,635 (236,841–292,429)	19.61 (17.55–21.67)
Men aged 35–50	376,715 (335,829–417,601)	27.91 (24.88–30.94)
Men aged 51–64	269,952 (233,053–306,850)	20.00 (17.27–22.74)
Men aged 65–80	165,584 (141,364–189,804)	12.27 (10.48–14.06)
Women		
Women aged 15–18	60,061 (52,104–68,017)	4.45 (3.86–5.04)
Women aged 19–24	109,339 (95,686–122,992)	8.10 (7.09–9.11)
Women aged 25–34	195,755 (169,636–221,875)	14.51 (12.57–16.44)
Women aged 35–50	279,713 (239,132–320,295)	20.73 (17.72–23.73)
Women aged 51–64	191,175 (149,042–206,201)	16.95 (14.17–19.74)
Women aged 65–80	132,554 (126,389–138,720)	13.16 (11.04–15.28)
Total	2,370,715 (2,062,730–2,678,700)	175.67 (152.85–198.49)

Estimates for the 2017 German population, aged 15 to 80 y.

We sought parameters on the direct relationship of food labels and added-sugar consumption. The estimates were evidence informed from a recent meta-analysis of the studies evaluating the effect of food labeling on energy intake since interventional studies examining exclusively the effect on the consumption of added sugar were not found (Shangguan et al. 2018). We used the effect on calories intake and modeled a reduction of 6.6% in added-sugar consumption, with no heterogeneity by age, sex, race, or socioeconomic status. We assumed the intervention would have a constant effect size over 10 y. Uncertainties around this parameter were assessed through deterministic sensitivity analysis.

The relationship between the amount of added sugar consumed and caries incidence was estimated based on a 11-y-long longitudinal study (Bernabe et al. 2016). Further details are provided in the Appendix.

DALYs for severe caries-related pain were used to express the burden of disease. The proportion, duration, and disability weight of symptomatic caries were derived from currently available literature (see Appendix for details).

The economic burden of caries, besides treatment costs, includes productivity losses of a person experiencing disease as well as productivity losses of caregivers. To estimate this indirect disease cost, we employed the WHO’s Commission on Macroeconomics and Health approach (Sachs 2001). The abovementioned estimates of DALYs were multiplied by 2017 gross domestic product (GDP) per capita value for Germany (International Monetary Fund [IMF] 2018). To provide a tentative estimate of the implementation costs of FoPFL, we used an approach previously employed by the Commission of the European Communities (2008; see Appendix for details).

### Analyses

Impacts on caries increment, dental treatment costs, DALYs, and productivity losses were estimated in a microsimulation model containing 500,000 gender-specific individuals for each abovementioned age category. To arrive at the population-level estimates, person-level impacts were multiplied by the population size for each age category (Appendix).

To assess the uncertainty in the effectiveness of FoPFL, we performed a deterministic sensitivity analysis embedding the lower and upper value (−4.4 and −8.8) of the reported meta-estimates (Shangguan et al. 2018). We also varied the cost input values to check the impact of potentially higher treatment costs due to copayments and restoration failures. Finally, we performed a probabilistic sensitivity analysis using 2 age- and gender-specific groups with the highest (boys aged 15 to 18 y) and lowest sugar consumption (women aged 51 to 64 y) within the German population and considering uncertainty in several input parameters with normal distribution (caries incidence, food labeling effect, relationship between the amount of sugar consumed and caries incidence, cost of restoration). Further details about input parameters, data sources, and calculations are described in the Appendix. To determine the extent of the uncertainty, we simulated 500,000 individuals per run of 2,000 simulations.

Annual discount rates of 3% for both benefits and costs were applied according to the applicable recommendations (Appendix). All simulations and analyses were performed using R (version 3.6.2; R Foundation for Statistical Computing).

A web-based dissemination tool was developed that facilitates online emulation of the decision-analytical model (more details are provided in the Appendix).

## Results

FoPFL in Germany over the period from 2017 to 2027 was estimated to prevent 2,370,715 (95% confidence interval [CI], 2,062,730–2,678,700) caries lesions (Table). A larger effect size can be expected within the male population due to higher sugar consumption, with a reduction in caries incidence by 1,319,452 (95% CI, 1,165,954–1,472,949) compared to 1,051,263 (95% CI, 896,775–1,205,751) caries lesions prevented within the female population. On a person level, men aged 15 to 18 y can be expected to benefit the most. Under conservative model assumptions, this would imply a hypothetical treatment cost saving of €175.67 million (95% CI, 152.85–198.49). If considering additional patient copayments (Appendix Table 2), treatment cost savings were €280.01 million (95% CI, 243.63–316.38); if considering restoration failures and consequences (Appendix Table 3), treatment costs savings were €352.60 million (95% CI, 306.79–398.40). In addition, FoPFL was estimated to avert 677.62 (95% CI, 589.59–765.65) DALYs. This translates into a reduction of €27.33 million (95% CI, 23.78–30.88) in productivity losses over 10 y. FoPFL was estimated to incur costs between €5.29 and €9.56 million, excluding the cost of relabeling and the marketing campaign.

**Figure 2.**
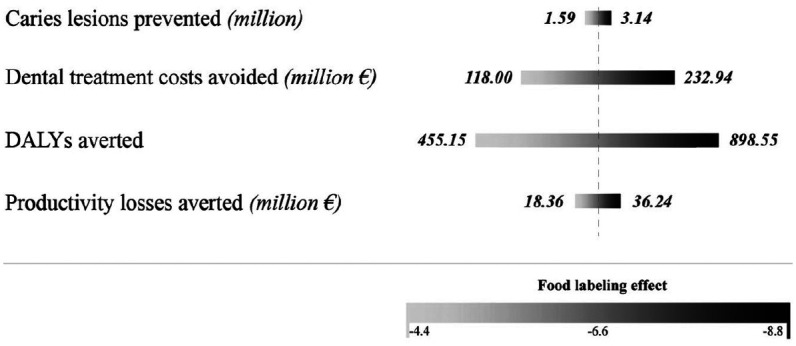
Oral health and economic benefits due to food labeling, 10-y time horizon. The bars indicate the outcomes for different input values used for food labeling effect (meta-estimates –6.6; 95% CI: −4.4 to −8.8). DALY, disability-adjusted life year.

The deterministic sensitivity analysis (Fig. 2) indicates that the estimated magnitude of caries increment reduction varied from 1,592,392 (95% CI, 1,284,147–1,900,637) to 3,143,636 (95% CI, 2,835,914–3,451,359) when lower and upper bounds (−4.4% and −8.8%, respectively) of food labeling effectiveness were used. The corresponding treatment cost savings are estimated to range between €118.00 million (95% CI, €95.16–€140.84) and €232.94 million (95% CI, €210.14–€255.75) (Fig. 2). The modeling process estimated that a total of 455.15 DALYs (95% CI, 367.05–543.26) would be averted when the effect of food labeling was reduced to 4.4%, relating to €18.36 million (95% CI, €14.80–€21.91) of productivity losses avoided. In case of the maximum reducing effect of 8.8%, 898.55 DALYs (95% CI, 810.59–986.50) were averted with a net saving of €36.24 million (95% CI, €32.69–€39.78) in terms of lost productivity.

**Figure 3.**
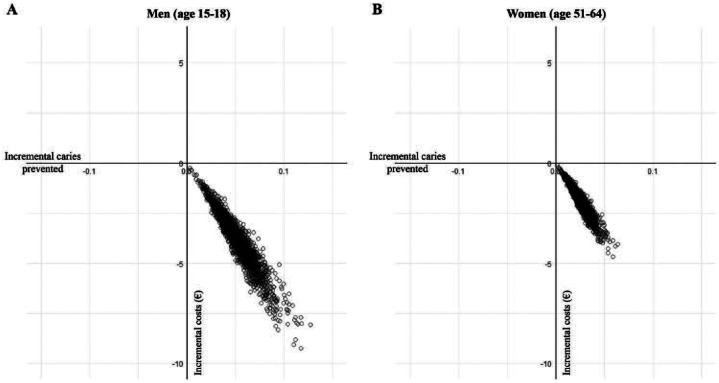
Probabilistic sensitivity analysis: scatterplot of incremental costs and effects of food labeling. Due to unavailable SD for the cost of restoration we used SD as ±20% of the mean reported value.

The results of the probabilistic sensitivity analysis are visually represented in a cost-effectiveness plane (Fig. 3). The plane is divided in 4 quadrants by the horizontal axis (the incremental effectiveness of FoPFL) and the vertical axis (incremental costs of FoPFL), with each of them having different implications for decision makers. The bottom-right quadrant is considered preferable, with positive oral health effects and cost-savings. Of the 2,000 probabilistic sensitivity analysis runs, with 500,000 individuals per run, 100% of the simulations were in the bottom-right quadrant (Fig. 3A, B).

For further robustness checks, the interested reader is referred to the web-based dissemination tool (Appendix).

## Discussion

Our findings show that FoPFL has the potential to substantially reduce caries increment and caries-related economic burden. Over a 10-y time horizon and using Germany as an example, nearly 2.4 million caries lesions could be prevented alongside reduced treatment costs between €176 million (lower bound) and €353 million (upper bound). On a person level, boys aged 15 to 18 y would have the greatest benefit.

Our study suggests that FoPFL reduces sugar consumption, generates oral health gains, and reduces dental treatment costs in a range between 1.6% and 3.2%. For comparison, implementing an SSB tax in Germany was previously estimated to reduce caries increment by 0.9% and associated treatment costs by 3% over the same time horizon (Schwendicke et al. 2016). This suggests that the impact of FoPFL may be on an order of magnitude that may not be smaller than that of SSB taxation.

This study has highly relevant implications for policy makers who are interested in food policies for oral health promotion and related economic benefits. To our knowledge, this is the first study that has focused on oral health and examined the effects food labeling. Previous literature has been mostly focused on the impacts of SSB taxation (Schwendicke et al. 2016; Briggs et al. 2017; Jevdjevic et al. 2019; Sowa et al. 2019). In addition, workplace bans on SSB sales have recently been proposed as another potential intervention to tackle sugar consumption and to reduce caries (Basu et al. 2020; Listl and Jevdjevic 2020).

The potential benefits of FoPFL are highly dependent on the response of consumers and the industry. The Federal Ministry for Food and Agriculture of Germany has recently expanded the nutritional labeling policy by introducing the Nutri-Score model that is expected to be implemented in 2020, albeit as a voluntary scheme (BMEL 2019). In the absence of specific evidence for the effect size of food labeling on sugar consumption, the effect of food labeling was conservatively proxied by meta-estimates based on 60 intervention studies that examined the intake of calories (Shangguan et al. 2018). No heterogeneity was found for the effect sizes of different types of food labels. While the exact effect size of food labeling on the consumption of foods with added sugar remains unclear, the Nutri-Score scheme was found to have greater effects on German consumers’ behavior than other types of food labels (Egnell et al. 2018). Considering that Nutri-Score was recently reported to effectuate a 10.6% improvement in food choices away from sugar-containing products (Egnell et al. 2020), and such an effect size is larger than assumed in our model, our findings might be considered lower-bound estimates for the impact of Nutri-Score on reducing sugar-attributable caries burdens.

Given the uncertainty of industry responses, several scenarios should be considered. The labeling policy could lead to the reformulation of sugar-containing products, resulting in even larger reductions in sugar intake. In that case, the real benefits might be even greater. Conversely, the industry might strategically use the labeling policy and impose a premium price on healthful products. This could negatively affect people with low socioeconomic background, attenuate positive health effects of labeling within this group, and increase already existent (oral) health inequalities (Kelly and Jewel 2019).

The present study has several strengths. It represents a timely assessment of the food labeling policy, at a time when there is an emerging need to address the sugar-related burden through a set of policies and there is increasing awareness for oral health on the global public health agenda (Watt et al. 2019). The decision-analytical microsimulation model was based on the age- and sex-specific data (added sugar consumption, caries incidence) for Germany. The uncertainties in model parameters were quantified through deterministic and probabilistic sensitivity analyses. Within the limitations of the simulation model, our study enables comparisons of oral health estimates with impacts of sugar-related food labeling on other health outcomes. Not least, the web-based dissemination tool (see Appendix for details) may be useful to raise awareness for the oral health–related relevance and potential implications of FoPFL.

Some potential limitations should also be mentioned. First, it was assumed that there would be only 1 surface restoration as a treatment option for each caries lesion predicted over 10 y. We did not take account of the number of surfaces before versus after caries occurrence or potentially differential carious attack rates following previous caries exposure. More complex and costly treatments were deliberately not evaluated, and such a simplifying approach is in line with ISPOR recommendations for economic evaluations (Caro et al. 2012). Second, disabilities resulting from the long-term consequences of caries such as tooth loss were not considered. This could lead to an underestimation of DALYs and productivity losses. Third, even though the cost of implementing FoPFL of added sugar was estimated to incur costs between €5.29 and €9.56 million, we did not account for the cost associated with relabeling that would be borne by the industry or the cost of the marketing campaigns. This was due to the absence of respective data for the specific context. These costs are generally relevant from a societal perspective (Commission of the European Communities 2008), particularly when estimating and comparing the cost-effectiveness of multiple alternative interventions. For instance, Sacks et al. (2011) found that traffic-light nutrition labeling and junk-food taxation would both be cost-saving interventions in preventing obesity in Australia, but food labeling was expected to incur 5 times greater implementation costs compared to taxation. Similarly, previous estimates from the US Food and Drug Administration (FDA) suggest that the implementation of changes in food labeling has the potential to produce net benefits, that is, cost savings exceed foregone benefits (FDA 2018). Fourth, our simulations assumed average effects of food labeling across all population groups, but it is unclear whether people with different SES background are similarly responsive to food labeling. If people with lower SES background have comparably low health literacy, food labeling may be more effective among people with high SES than people with lower SES background. Finally, we used data from Germany, and the question can be raised whether the results are also generalizable to other countries. Also, note that in settings where treatment costs are largely covered by publicly financed insurance, such as Germany, consumers may be less inclined to behavior change because of consumer moral hazard (Allcott et al. 2019).

Before choosing a preferred strategy to achieve lower sugar consumption, policy makers should use available evidence and carefully consider all the context-specific factors as well as the intervention-related benefits and costs. If appropriate, food labeling can be implemented as a stand-alone strategy, or it can be part of a more multifaceted approach—for example, complementing SSB taxation as in Chile and Mexico (Taillie et al. 2020; White and Barquera 2020), marketing restrictions in Sweden (Mazzocchi 2017), or workplace bans on SSB sales as recently proposed in a US context (Basu et al. 2020; Listl and Jevdjevic 2020). More generally, the formulation and implementation of sensible diet intervention options to improve health and save costs continues to be a multifaceted issue with pros and cons for the various alternative routes of action, which require collective problem solving and, ultimately, deliberate political choices (Cleghorn et al. 2019).

In conclusion, our study suggests that implementing FoPFL has the potential to improve oral health and yield substantial economic benefits. However, the magnitude of the health and economic gains is highly dependent on the responses by consumers and the food industry. The context-specific factors and implementation costs should also be taken into account. In order to be successful, implementation or alteration of FoPFL strategies therefore requires careful planning and monitoring.

## Author Contributions

M. Jevdjevic, contributed to conception, design, data acquisition, analysis, and interpretation, drafted and critically revised the manuscript; S.R.W. Wijn, contributed to data acquisition, analysis, and interpretation, critically revised the manuscript; A.L. Trescher, R. Nair, M. Rovers, contributed to data acquisition and interpretation, critically revised the manuscript; S. Listl, contributed to design, data acquisition, and interpretation, critically revised the manuscript. All authors gave final approval and agree to be accountable for all aspects of the work.

## Supplemental Material

sj-pdf-1-jdr-10.1177_0022034520979147 – Supplemental material for Front-of-Package Food Labeling to Reduce Caries: Economic Evaluation Click here for additional data file. Supplemental material, sj-pdf-1-jdr-10.1177_0022034520979147 for Front-of-Package Food Labeling to Reduce Caries: Economic Evaluation by M. Jevdjevic, S.R.W. Wijn, A.L. Trescher, R. Nair, M. Rovers and S. Listl in Journal of Dental Research
