# Supplementary material for: Front-of-Package Food Labeling to Reduce Caries: Economic Evaluation
Source: J Dent Res. 2020 Dec 17;100(5):472–8. doi: 10.1177/0022034520979147 (PMC8058828; doi:10.1177/0022034520979147)
Supplement: sj-pdf-1-jdr-10.1177_0022034520979147 – Supplemental material for Front-of-Package Food Labeling to Reduce Caries: Economic Evaluation [file sj-pdf-1-jdr-10.1177_0022034520979147.pdf]

APPENDIX MATERIAL: Front-of-package food labeling to reduce caries: economic evaluation

M Jevdjevic [a] , SRW Wijn [b], AL Trescher [c], R Nair [a] , M Rovers [b,d], S Listl [a,c]

[a] Department of Dentistry - Quality and Safety of Oral Healthcare, Radboud University Medical Center, Radboud Institute for Health Sciences, Nijmegen, The Netherlands

[b] Department of Operating Rooms, Radboud UMC, Nijmegen, The Netherlands

[c] Department of Conservative Dentistry, Translational Health Economics Group, Heidelberg University, Heidelberg, Germany

[d] Department of Health Evidence, Radboud UMC, Nijmegen, The Netherlands

1. Further background information

Food labeling is a frequently proposed strategy for reducing the amount of sugar intake (Kanter et al. 2018). It seeks to convert nutritional information into informed consumer choices towards healthy food and beverage consumption. Relevant prerequisites for food labeling to be effective are sufficient health literacy, i.e. the consumer is able to acquire and interpret health-related information, and self-efficacy, i.e. the consumer beliefs in a healthy diet and is self-confident to achieve it (Cha et al. 2014). To date, there are several labeling schemes employed, varying in presentation and types of information displayed. When making healthier food choices, the most important are those providing the nutrition content. A variety of formats has been used from presenting the amount of nutrients as a proportion of recommended daily intake, to the usage of symbols, words and colors to evaluate overall healthiness of a product. In addition, they can include positive or negative signposting. As such, label formats may also contribute to the differences in individuals' utilization of nutrition information. For example, with the Nutri-Score, the Traffic Light or the Nordic Keyhole it is sufficient to understand the meanings of colors, logos or symbols, while to select healthier foods with the Reference Intakes, numeracy and literacy skills are required (Cha et al. 2014; Jones et al. 2019). On the other hand, the effects of pricing to reduce the amount of sugar intake (e.g. SSB taxation) are based on the acquisitive power of consumers. Increasing prices of particular unhealthy products would reduce their affordability (e.g. SSB taxation), and it is expected to cause higher demand responses among low-income consumers, assuming that the prices of all other goods and acquisitive power remain equal (Allcott et al. 2019).

Currently, available evidence suggests that there is a relationship between socioeconomic gradient and diet quality. Less affluent groups have a diet of poor quality, with high sugar consumption being one of its most dominant contributors, whereas groups with a high socioeconomic status tend to consume less sugar (Darmon and Drewnowski 2008). In addition, several studies reported that people with lower education demonstrated lower health literacy than people with higher education (Lee et al. 2010; Nutbeam 2008; Van Der Heide et al. 2013).

Most common alternatives for added-sugars are sweeteners and fruits. Various artificial sweeteners have been commonly used as an alternative to high calorie sugar. However, even being often low on calories and cheaper alternative, they are not always teeth-friendly. According to Wiggins et al. (2015), less calorie dense foods, fruits among them, also imply higher costs on consumers. Therefore, purchasing power might still be a limiting factor for changes in purchasing behaviors despite the efforts of FoPFL.

2. Parameter estimation and uncertainties

- Added-sugar consumption

Baseline intake of free sugars was obtained from the German National Nutrition Survey II (NVS II). Data were available for the population aged 14 to 79 (Appendix Table 1), stratified for gender and age groups (15-18, 19-24, 25-34, 35-50, 51-64, 65-80 years old) (Heuer 2018).

Appendix Table 1. Added-sugar consumption of the German population stratified by age gender groups, grams per day (Heuer 2018)

Age	Men	Women
15 - 18	102	74
19 - 24	102	79
25 - 34	92	72
35 - 50	84	62
51 - 64	61	50
65 - 80	55	51

- Relationship between the amount of added-sugar consumed and caries incidence:

Bernabe et al. (2016) have found that "Over 11 years Decayed, Missing, Filled Teeth (DMFT) score increases by 0.1 units for every 10 grams of sugar consumed per day". In our study, we assumed linear caries development over the 11-year time horizon. Therefore, the mean annual increase in DMFT amounts to 0.0091 for every 10 grams consumed per day. It was assumed that the DMFT increment solely presents yearly caries incidence (Kassebaum et al. 2015), and this estimate was converted into the reduction of probability for caries development.

0.009587 ([lower bound] 0.003633; [upper bound] 0.013547) DMFT annually for every 10 grams of sugar consumed per day (Bernabe et al. 2016).

- Caries incidence

A person-level yearly caries incidence with 95% confidence interval, stratified for age and gender was derived from the publicly available online platform of The Institute for Health Metrics and Evaluation (IHME 2019). A yearly probability for caries development was then established based on the following formula: $1 - e^{(-YCI)}$, with YCI being person-level yearly caries incidence.

- Caries-related Disability Adjusted Life Years (DALYs)

The caries burden due to morbidity was estimated through DALYs based on the Global Burden of Disease (GBD) methodology (Bernabe et al. 2020). In our analysis, we accounted only for DALYs resulting from severe pain due to caries. According to the GBD estimates, the proportion of symptomatic caries causing severe pain was 18.9%. On average, the duration of severe toothache was 55.2 days (0.15 year). The disability weight of symptomatic caries was 0.010. To arrive at the final estimates the total number of caries lesions was multiplied by the likelihood of experiencing severe toothache (0.189), duration of toothache (0.15 year), and disability weight (0.010).

- Treatment costs

Since about 90% of the German population is covered by statutory health insurance (GKV) (Bundesgesundheitsministerium 2020), we included the costs for vitality test, anaesthesia, and a composite filling for both publicly insured (90% of cases; reimbursement according to BEMA-Z: €73.11; BEMA positions 8, 40 & 13e; no patient co-payment) and privately insured patients (10% of cases; reimbursement according to GOZ @ multiplication factor 2.3: €83,03; GOZ positions 0070, 0090, vm113 [Ultracain D-S] & 2060) such that the average treatment cost amounts to €74.10 (Kassenzahnärztliche Bundesvereinigung 2018; Kassenzahnärztliche Bundesvereinigung 2016).

Sensitivity analyses

Given that higher treatment costs are not unusual in Germany due to the possibility of patient co-payments for publicly insured patients and a higher than the average multiplication factor of 2.3 being applied for privately insured patients (depending on treatment complexity for the individual patient), treatment costs of a patient under statutory health insurance were modelled. As a reference case, we used a patient who is treated at Heidelberg University Hospital where an additional patient co-payment of €45 is issued for a 1-surface filling; hence the total treatment costs amount to €118.11 in our sensitivity analysis. The results from this sensitivity analysis are shown in Appendix Table 2 below.

To arrive at the population level estimates, person-level impacts were multiplied by the population size for each age category (Federal Statistical Office 2019). Annual discount rates of 3% for both benefits and costs were applied according to the applicable recommendations (IQWiG 2019).

Appendix Table 2. Sensitivity analysis – Patient co-payment included; Number of prevented caries lesions and treatment costs avoided due to food labeling with 95% Confidence Intervals, 10-year time horizon

	**Caries lesions prevented (total)**	**Treatment costs avoided (million €)**
**Men**		
Men aged 15-18	89,638 (80,984 to 98,292)	10.59 (9.57 to 11.61)
Men aged 19-24	152,929 (137,883 to 167,974)	18.06 (16.29 to 19.84)
Men aged 25-34	264,635 (236,841 to 292,429)	31.26 (27.97 to 34.54)
Men aged 35-50	376,715 (335,829 to 417,601)	44.49 (39.66 to 49.32)
Men aged 51-64	269,952 (233,053 to 306,850)	31.88 (27.53 to 36.24)
Men aged 65-80	165,584 (141,364 to 189,804)	19.56 (16.70 to 22.42)
**Women**		
Women aged 15-18	60,061 (52,104 to 68,017)	7.09 (6.15 to 8.03)
Women aged 19-24	109,339 (95,686 to 122,992)	12.91 (11.30 to 14.53)
Women aged 25-34	195,755 (169,636 to 221,875)	23.12 (20.04 to 26.21)
Women aged 35-50	279,713 (239,132 to 320,295)	33.04 (28.24 to 37.83)
Women aged 51-64	191,175 (149,042 to 206,201)	27.02 (22.58 to 31.46)
Women aged 65-80	132,554 (126,389 to 138,720)	20.98 (17.60 to 24.35)
**Total**	2,370,715 (2,062,730 to 2,678,700)	280.01 (243.63 to 316.38)

To demonstrate potential implications of restoration failure and subsequent treatment modalities within the restorative cycle (restoration renewal, endodontic treatment, and implant placement), an additional sensitivity analysis was conducted. An annual failure rate of 2.4% per year was applied to all restorations placed within 10-year horizon (Opdam et al. 2014). We assumed 33% probability for each alternative treatment: New restoration, €74.1; Endodontic treatment, €1703 EUR (Schwendicke and Göstemeyer 2016); Implant, €2050 (Pretzl et al. 2009). Based on this simplifying assumption, an average estimated treatment cost would amount to €1276 per case of initial restoration failure. When accounting for 2.4% failure rate, additional treatment costs sum up to €30.62 on average. We integrated the cost of initial restoration, patient co-payment and additional treatment costs. Finally, the value of €148.73 was

used as input. The results from this sensitivity analysis are shown in Appendix Table 3 below. Finally, for probabilistic sensitivity analysis, due to unavailable standard deviation (sd) for the cost of restoration, we used the confidence interval as +/-20% of the mean reported value (€74.10).

Appendix Table 3. Sensitivity analysis – Patient co-payment and full restorative cycle included; Number of prevented caries lesions and treatment costs avoided due to food labeling with 95% Confidence Intervals, 10-year time horizon

	**Caries lesions prevented (total)**	**Treatment costs avoided (million €)**
**Men**		
Men aged 15-18	89,638 (80,984 to 98,292)	13.33 (12.04 to 14.62)
Men aged 19-24	152,929 (137,883 to 167,974)	22.75 (20.51 to 24.98)
Men aged 25-34	264,635 (236,841 to 292,429)	39.36 (35.23 to 43.49)
Men aged 35-50	376,715 (335,829 to 417,601)	56.03 (49.95 to 62.11)
Men aged 51-64	269,952 (233,053 to 306,850)	40.15 (34.66 to 45.64)
Men aged 65-80	165,584 (141,364 to 189,804)	24.63 (21.03 to 28.23)
**Women**		
Women aged 15-18	60,061 (52,104 to 68,017)	8.93 (7.75 to 10.12)
Women aged 19-24	109,339 (95,686 to 122,992)	16.26 (14.23 to 18.29)
Women aged 25-34	195,755 (169,636 to 221,875)	29.11 (25.23 to 33.00)
Women aged 35-50	279,713 (239,132 to 320,295)	41.60 (35.57 to 47.64)
Women aged 51-64	191,175 (149,042 to 206,201)	34.03 (28.43 to 39.62)
Women aged 65-80	132,554 (126,389 to 138,720)	26.42 (22.17 to 30.67)
**Total**	2,370,715 (2,062,730 to 2,678,700)	352.60 (306.79 to 398.4)

- Cost of intervention

To provide a range of possible costs associated with nutrition labeling we used the approach previously employed by The Commission of the European Communities (2008). In The Impact Assessment Report on General Food Labeling Issues, they accounted for the cost of familiarization with new regulations, collection of the necessary information to be presented on the label, label re-design and administration. The cost of familiarizing with new policy was estimated at €1408 per company (the baseline year 2004). For our analysis, we assumed the same cost, amounting to €1741 when adjusted for inflation rates to the 2017 price year. According to GTAI (2019), there were 6000 companies within the food industry in Germany in 2017. Therefore, the familiarization with new regulations would incur costs of €5.22 million. We assumed food producers to be already familiar with the content of their products and the necessary information to be displayed on front-of-package (FOP) labels. Due to the lack of setting-specific data on re-labeling costs we were not able to take this into account. However, more than 80% of companies would change their labels over a 3-year period irrespectively of food labeling interventions (Rabinovich et al. 2008). The overall administration-related burden due to general food labeling legislation ranges between 0.01-0.69% of the food industry revenues, where nutrition labeling accounts for 4% of it (The Commission of the European Communities 2008). In 2017, the food industry revenues for added-sugar containing products were as follows: 1) €11.9 billion for confectionery and snacks (chocolate products, sugar products, fine baked goods, snacks, cocoa and chocolate semi-finished products, ice-cream, raw mixtures); 2) €6.7 billion for non-alcoholic beverages (€2.7 billion for water) (GTAI 2019). Administrative costs due to nutrition labeling of added-sugar containing products amounts between €64,800 and € 4,341,600. In total, considering currently available evidence, the implementation of FOPFL of added-sugar was estimated to incur costs between €5.29 and €9.56 million, excluding the cost of relabeling.

3. Interactive web-based dissemination tool

The interactive tool, displayed in Figure 1, will calculate the individual-level effects in caries lesions and costs per person over 10 years given the values set in the tool. Additionally, the tool will calculate

the total caries lesions prevented and treatment costs avoided by simulating all the age categories and multiplying the estimates by the size of the selected population group.

The tool was built using the shiny (version 1.4.0) and shinydashboard (version 0.7.1) package (Chang and Borges Ribeiro 2018; Chang 2019). The online dissemination tool is available here: https://stanwijn.shinyapps.io/FoodLabeling-CariesPrevention .

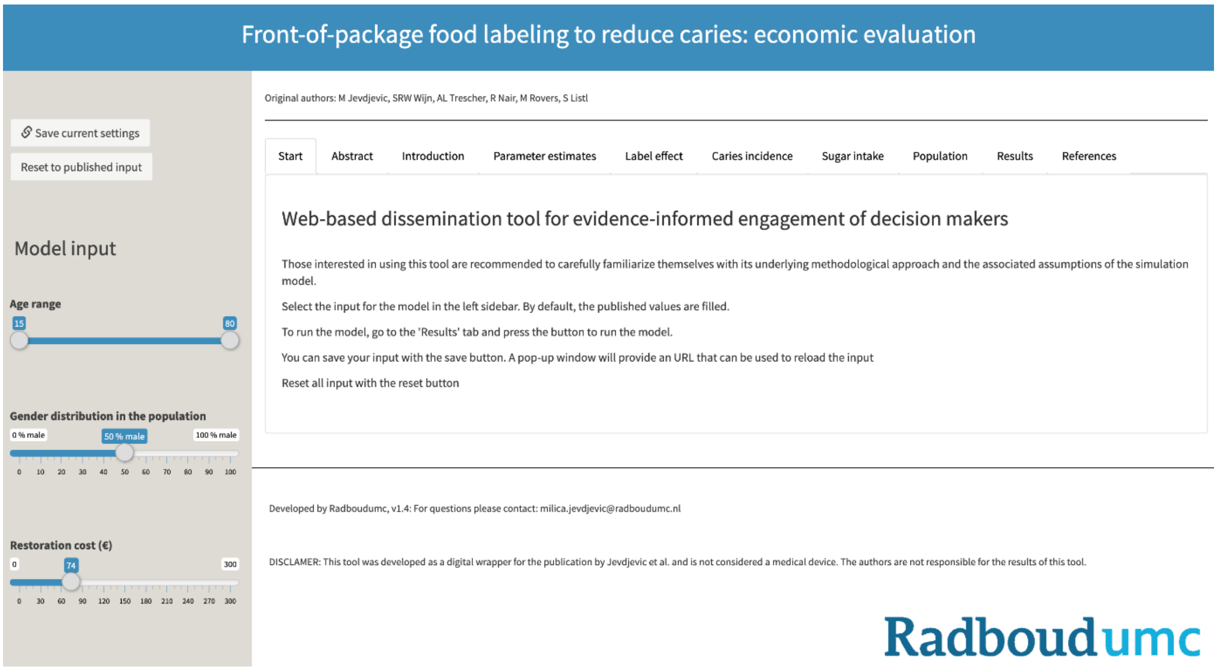

Figure 1. Screenshot of the online dissemination tool, accessible via https://stanwijn.shinyapps.io/FoodLabeling-CariesPrevention/

References:

Allcott H, Lockwood BB, Taubinsky D. 2019. Should we tax sugar-sweetened beverages? An overview of theory and evidence. Journal of Economic Perspectives. 33(3):202-227.

Bernabe E, Marcenes W, Hernandez CR, Bailey J, Abreu LG, Alipour V, Amini S, Arabloo J, Arefi Z, Arora A et al. 2020. Global, regional, and national levels and trends in burden of oral conditions from 1990 to 2017: A systematic analysis for the global burden of disease 2017 study. J Dent Res. 99(4):362-373.

Bernabe E, Vehkalahti MM, Sheiham A, Lundqvist A, Suominen AL. 2016. The shape of the dose-response relationship between sugars and caries in adults. J Dent Res. 95(2):167-172.

Bundesgesundheitsministerium. 2020. Krankenversicherung – versicherte in der gkv, https://www.Bundesgesundheitsministerium.De/themen/krankenversicherung/online-ratgeber-krankenversicherung/krankenversicherung.Html, [accessed on 30.07.2020.].

Cha E, Kim KH, Lerner HM, Dawkins CR, Bello MK, Umpierrez G, Dunbar SB. 2014. Health literacy, self-efficacy, food label use, and diet in young adults. American journal of health behavior. 38(3):331-339.

Chang and Borges Ribeiro. 2018. Shinydashboard: Create dashboards with 'shiny'. R package version 0.7.1. Https://cran.R-project.Org/package=shinydashboard.

Chang C, Allaire,Xie and McPherson 2019. Shiny: Web application framework for r. R package version 1.4.0. Https://cran.R-project.Org/package=shiny.

Darmon N, Drewnowski A. 2008. Does social class predict diet quality? The American Journal of Clinical Nutrition. 87(5):1107-1117.

Federal Statistical Office 2019. Wiesbaden, https://www.Destatis.De/genesis/online/data;sid=796ddeed1e2a8edba6b3c7e5c70df83b.Go_2_1?Operation=abruftabellebearbeiten&levelindex=2&levelid=1557842151267&auswahloperation=abruftabelleauspraegungauswaehlen&auswahlverzeichnis=ordnungsstruktur&auswahlziel=werteabruf&selectionname=12411-0006&auswahltext=&nummer=5&variable=5&name=ges&werteabruf=value+retrieval, [accessed on 14.05.2019.].

GTAI. 2019. Germany trade and invest, the food & beverage industry in germany, https://www.Gtai.De/resource/blob/64004/e80f4dd7ccd691158b0ee2bc10f8cd6c/industry-overview-food-beverage-industry-en-data.Pdf, [accessed on 25.07.2020.].

Heuer T. 2018. Zuckerkonsum in deutschland. Aktuelle Ernährungsmedizin. 43(S 01):S8-S11.

IHME. 2019. Institute for health metrics and evaluation, global health data exchange: Gbd results tool [internet]. Http://ghdx.Healthdata.Org/gbd-results-tool, [accessed on 10.05.2019.].

IQWiG. 2019. Accessed on 26.02.2020.

Jones A, Neal B, Reeve B, Ni Mhurchu C, Thow AM. 2019. Front-of-pack nutrition labelling to promote healthier diets: Current practice and opportunities to strengthen regulation worldwide. BMJ Glob Health. 4(6):e001882.

Kanter R, Vanderlee L, Vandevijvere S. 2018. Front-of-package nutrition labelling policy: Global progress and future directions. Public health nutrition. 21(8):1399-1408.

Kassebaum NJ, Bernabe E, Dahiya M, Bhandari B, Murray CJ, Marcenes W. 2015. Global burden of untreated caries: A systematic review and metaregression. J Dent Res. 94(5):650-658.

Kassenzahnärztliche Bundesvereinigung 2018. Jahrbuch 2018. Cologne.

Kassenzahnärztliche Bundesvereinigung 2016. Einheitlicher bewertungsmaßstab für zahnärztliche leistungen gemäß § 87 abs. 2 und 2h sgb v "bema.

Lee S-YD, Tsai T-I, Tsai Y-W, Kuo KN. 2010. Health literacy, health status, and healthcare utilization of taiwanese adults: Results from a national survey. BMC public health. 10(1):614.

Nutbeam D. 2008. The evolving concept of health literacy. Social science & medicine. 67(12):2072-2078.

Opdam N, Van De Sande F, Bronkhorst E, Cenci M, Bottenberg P, Pallesen U, Gaengler P, Lindberg A, Huysmans M, Van Dijken J. 2014. Longevity of posterior composite restorations: A systematic review and meta-analysis. Journal of dental research. 93(10):943-949.

Pretzl B, Wiedemann D, Cosgarea R, Kaltschmitt J, Kim TS, Staehle HJ, Eickholz P. 2009. Effort and costs of tooth preservation in supportive periodontal treatment in a german population. Journal of Clinical Periodontology. 36(8):669-676.
Rabinovich L, Tiessen J, Tsang F, Van Stolk C. 2008. Assessing the impact of revisions to the eu nutrition labelling legislation.
Schwendicke F, Göstemeyer G. 2016. Cost-effectiveness of single-versus multistep root canal treatment. Journal of endodontics. 42(10):1446-1452.
The Commission of the European Communities. 2008. Impact assessment report on general food labelling issues. Https://ec.Europa.Eu/food/sites/food/files/safety/docs/labelling_legislation_general-food-labelling_en.Pdf, [accessed on 22.07.2020].
Van Der Heide I, Wang J, Droomers M, Spreeuwenberg P, Rademakers J, Uiters E. 2013. The relationship between health, education, and health literacy: Results from the dutch adult literacy and life skills survey. Journal of health communication. 18(sup1):172-184.
Wiggins S, Keats S, Han E. 2015. The rising cost of a healthy diet. Changing relative prices of foods in high-income and emerging economies London: Overseas Development Institute.
